# Evaluation of synergistic/antagonistic antibacterial activities of fatty oils from apricot, date, grape, and black seeds

**DOI:** 10.1038/s41598-024-54850-y

**Published:** 2024-03-19

**Authors:** Farah M. Joujou, Nada El Darra, Hiba N. Rajha, Elie Salem Sokhn, Nisreen Alwan

**Affiliations:** 1 Department of Nutrition and Dietetics, Faculty of Health Sciences, Beirut Arab University, Tarik El Jedidah, Riad El Solh, P.O. Box 115020, Beirut, 1107 2809 Lebanon; 2 Département de Génie Chimique et Pétrochimique, Faculté d’Ingénierie, Ecole Supérieure, d’Ingénieurs de Beyrouth (ESIB), Université Saint-Joseph de Beyrouth, CST Mkalles Mar, Rokos, Riad El Solh, Beirut, 1107 2050 Lebanon; 3 Molecular Testing Laboratory, Department of Medical Laboratory Technology, Faculty of Health Sciences, Beirut Arab University, P.O. Box 11-5020, Beirut, Lebanon; 4 Environmental and Public Health Department, College of Health Sciences, Abu Dhabi University, PO Box 59911, Abu Dhabi, UAE

**Keywords:** Microbiology, Antimicrobials, Antimicrobial resistance

## Abstract

The increasing antimicrobial resistance requires continuous investigation of new antimicrobial agents preferably derived from natural sources. New powerful antibacterial agents can be produced by simply combining oils that are known for their antibacterial activities. In this study, apricot seed oil (ASO), date seed oil (DSO), grape seed oil (GSO), and black seed oil (BSO) alone and in binary mixtures were assessed. Fatty acid profiles of individual oils and oil mixtures showed linoleic acid, oleic acid, palmitic acid, stearic acid, and linolenic acid contents. Linoleic acid was the most abundant fatty acid in all samples except for ASO, where oleic acid was the dominant one. GSO showed the highest total phenolic content while ASO showed the lowest one. Antibacterial screening was performed against *Escherichia coli*, *Klebsiella pneumoniae*, *Pseudomonas aeruginosa*, *Proteus mirabilis*, and *Staphylococcus aureus*. Results showed antibacterial activity in all oils against tested strains except for ASO against *S. aureus*. Highest antibacterial activity recorded was for ASO against *P. mirabilis*. ASO-GSO mixture (AG) was the best mixture where it showed synergistic interactions against all strains except *P. aeruginosa*. In conclusion, seed oil mixtures are likely to show promising antibacterial activities against specific strains.

## Introduction

Nowadays, studies aim to assess new edible seed oils recovered from plant sources^1,2^. Seed oils are mainly composed of triglycerides with other minor compounds including phenols, phytosterols, tocopherols, carotenoids, and phospholipids^1^. They are used in food, cosmetics, pharmaceutical, and chemical industries for different biological and technological purposes^3^.

Antimicrobial Resistance (AMR) is a major problem in public health, posing a potential threat to living organisms and causing death^4^. It happens as a consequence of antibiotics misuse without consulting healthcare and agricultural professionals^5,6^.

In this regard, plant extracts represent unlimited sources of bioactive chemicals that can be used as antimicrobial agents^7^. Oils represent an important source of natural antimicrobials allowing their implementation as natural antimicrobials in food^8,9^. For example, chitosan films enriched with fig seed oil alone or in combination with apricot and plum seed oils were found to protect against microbial spoilage when applied on fresh lemon and banana slices^9^.

*Prunus armeniaca* L., known as apricot, is a member of *Rosaceae* family^10^. Apricots are widely consumed and used in food manufacturing like juices and jams, which in turn produce large masses of seeds that need further exploitation^11^. Apricot seeds contain oil content up to 50% for each seed, making it possible to produce tons of ASO each year^12^. ASO is an edible oil and serves as a functional ingredient in different industries including the food sector^3^. Apricot seeds possess several pharmacological functions including antioxidant activity^13^, antitubercular activity, cardioprotective effect, and hepatoprotective effect^14^. In addition, seeds showed antibacterial and antifungal activities. Apricot seed extracts showed antimicrobial activity against *E. coli*, *P. mirabilis*, *S. aureus*, *Candida albicans*, *C. glabrata*, and *C. parapsilosis*^13^. Apricot seed essential oil showed an activity against *Bacillus cereus*, *Enterococcus faecalis*, *K. pneumoniae*, and *Serratia marcescens*^15^.

*Vitis vinifera*, known as grape, has a valuable nutritional content and important pharmaceutical properties of its derivatives such as seed and peel extracts^16^. The winemaking process produces about 0.2 kg of grape pomace for each kg of crushed grapes, where seeds represent about 25% of total pomace^17^. Grape seed is rich in cellulose, protein, bioactive compounds, and has up to 20% oil content^17,18^. GSO can be used for culinary purposes, such as frying food, and as an emulsifier in sauces and dressings production^19^. It can also be used as an ingredient in pharmaceutical, cosmetics, and biodiesel production^19^. GSO antibacterial activity was reported against *S. aureus* and *E. coli*^16^. Grape seed extracts showed antibacterial activity against *S. aureus*, *K. pneumoniae*, *P. aeruginosa*, and *E. coli*^20^ as well as antifungal activity^21^. Furthermore, an antioxidant activity, cardioprotective effect^16^, hepatoprotective effect^22^, neuroprotective effect^23^, and anti-inflammatory effect^24^ were also observed.

*Phoenix dactylifera*, known as date or date palm, is a nutritious fruit recording an annual increase in its production^25,26^. Date seed has a valuable chemical content; however, it is considered as an industrial waste that requires further exploitation^26^. Date seed has 5% to 13% oil content that can be an excellent ingredient in different industries due to its essential functions and edibility^26,27^. For example, DSO can be applied as a natural ingredient in UV protector formulas for its ability to absorb light in UV range, and as a natural colorant in butter and margarines for its yellow color^28^. Date seed extracts showed an important antibacterial activity against different strains including *E. coli*, *K. pneumoniae*, *P. mirabilis*, *P. aeruginosa*, *S. aureus*, *Bacillus subtilis*, and *Streptococcus pyogenes*^29,30^. In addition, DSO showed an antioxidant activity^31^, a chemopreventive effect^32^, and a photoprotective effect^33^.

*Nigella sativa*, an annual flowering plant belonging to *Ranunculaceae* family, produces numerous seeds commonly known as black seeds or black cumin. Black seed contains fixed oil, essential oil, protein, saponin, and alkaloid in addition to important nutritional components such as minerals, carbohydrates, fats, and vitamins^34^. The seed contains 30% to 44.2% fixed oil, which is extracted for several purposes such as culinary uses, food flavorings, food preservatives for confectioneries, fats stabilizers, and pharmaceutical and therapeutic applications^35^. Many studies were reported on the therapeutic applications of BSO. For example, antibacterial activity^36^, antifungal activity^34^, anti-inflammatory activity, anti-histaminic effect^35^, antioxidant activity^37^, antiviral effect^38^, anticancer activity^36^, gastroprotective effect^39^, and hepatoprotective effect were reported^34^.

This study focused on ASO, DSO, and GSO since these oils have limited studies on their antibacterial activity. BSO was also added for its well-known therapeutic potency^36^, making it of interest to test for possible synergism when combined with other oils.

Synergy improves biological properties where mixing oils can increase the components’ diversity and their structures’ complexity, which in turn provide multiple sites of actions and affect multiple biochemical processes in bacteria^40^. In addition, pathogens cannot develop resistance to multiple components present in the oil mixtures leading to an amplified antimicrobial activity^40^. The innovation of this study is screening for synergistic antibacterial activity of ASO, DSO, GSO, and BSO when they are applied in binary mixtures. Fatty acid profile and total phenol content of oils and their mixtures were evaluated as well. To our knowledge, no previous studies were done to test for possible synergism between these oils. Oils and oil mixtures with important antibacterial activity can be then tested for their ability to serve as natural antimicrobials when applied in films or nanoparticles in different food, pharmaceutical, and other manufactured products.

## Materials and methods

### Seed oils

Commercially available ASO, DSO, GSO, and BSO were bought from a local shop, originating from France. Oils were extracted from seeds by cold-pressing. All oils were filtered using sterile 0.22 µm pore syringe filters prior to experiments. Oil mixtures were prepared as follow: AB (ASO+BSO), AG (ASO+GSO), AD (ASO+DSO), BD (BSO+DSO), BG (BSO+GSO), and GD (GSO+DSO). All mixtures were prepared in 1:1 (v/v).

### Polyphenol extraction and quantification

Polyphenols were first extracted according to^41^. 2 mL of hexane were added to 1 gram of each oil, then 5 mL of 80% methanol/water solution were added. The mixture was vortexed and then centrifuged (5000 *g*, 20 min). Aqueous phase was analyzed.

Total phenolic content was measured by Folin–Ciocalteau method according to^42^. 1 mL of diluted Folin solution (1:10) was added to 0.2 mL of aqueous phase extracts. Then 0.8 mL of sodium carbonate solution (7.5%) was added. Solutions were incubated at room temperature for 1 h, and then absorbance was read at 765 nm. Samples were measured in triplicates and mean value was reported. Calibration curve of gallic acid was prepared, and results were expressed as mg GAE/g of oil.

### Fatty acid analysis

To determine fatty acid composition of oils and oil mixtures, fatty acid methyl esters were prepared by derivatization reaction using trimethylsulfonium hydroxide (TMSH)^43^. 20 μL of oil sample were dissolved in 2 mL of tert-butyl methyl ether. Then, 20 μL of this solution were removed and derivatized using 20 μL TMSH. Samples were incubated for 1 h, and then derivatized samples were measured using GC-MS (Shimadzu GCMS-QP2010 Plus)^44^. Helium was used as a carrier gas with split ratio 1:20 and flow rate 2.06 mL/min. Injection volume was 1 μL. Injection temperature and interface temperature were 230 °C. Scanning by MS was set at 35–250 m/z, ionization energy of 70 eV, and ion source temperature of 200 °C. Programming temperature was set as follow: 120 °C to 190 °C at 10 °C/min, 190 °C to 220 °C at 5 °C/min (hold 2 min).

### Antibacterial assessment

#### Bacterial strains

Five bacterial strains were used in the study due to their clinical significance. Four gram-negative strains including *E. coli* (ATCC 25922), three clinical isolates of *K. pneumoniae*, *P. aeruginosa*, *P. mirabilis*, and one gram-positive strain of *S. aureus* clinical isolate were provided from Beirut Arab University laboratory. Fresh cultures were prepared on blood agar and MacConkey agar incubated overnight at 37 °C.

#### Disk diffusion for single oils

Kirby–Bauer method was used to perform agar disk diffusion for the antibacterial screening of oils alone and in mixtures. Mueller Hinton agar plates were inoculated with bacterial suspensions with load of 1.5 × 10^8^ CFU per mL. 6 mm disks of Whatman filter paper were placed on the agar, and 20 µL of each oil were added on each disk^45^. Blank disks were used as a negative control. After incubating the plates at 37 °C for 16–18 h, diameters of inhibition zones (IZs) were measured. Each experiment was repeated three times. Results were reported as the mean of three trials.

#### Disk diffusion for oil mixtures

Same method was used to screen the synergistic antibacterial effect of mixtures. 20 µL of each mixture were added on each disk. Tests were repeated three times, and zones were reported as the mean of three trials.

Data were interpreted according to^46^ by comparing experimental IZs obtained from screening oil mixtures with theoretical sum of IZs of separate oils; interactions were interpreted as: synergy if experimental value is higher than the theoretical, antagonism if experimental is lower than theoretical, and indifference if theoretical is approximately equal to the experimental value. Theoretical IZs of mixtures were calculated according to^47^.

### Statistical analysis

Data in all figures were reported as mean ± standard deviation of three trials. Analysis of Variance (ANOVA) was conducted followed by post-hoc test to compare and check for significance between groups. Significance level was set at *p* < 0.05 for all tests. SPSS Statistics 20.0 was used to conduct statistical analysis.

## Results and discussion

### Fatty acid composition

**Figure 1:**
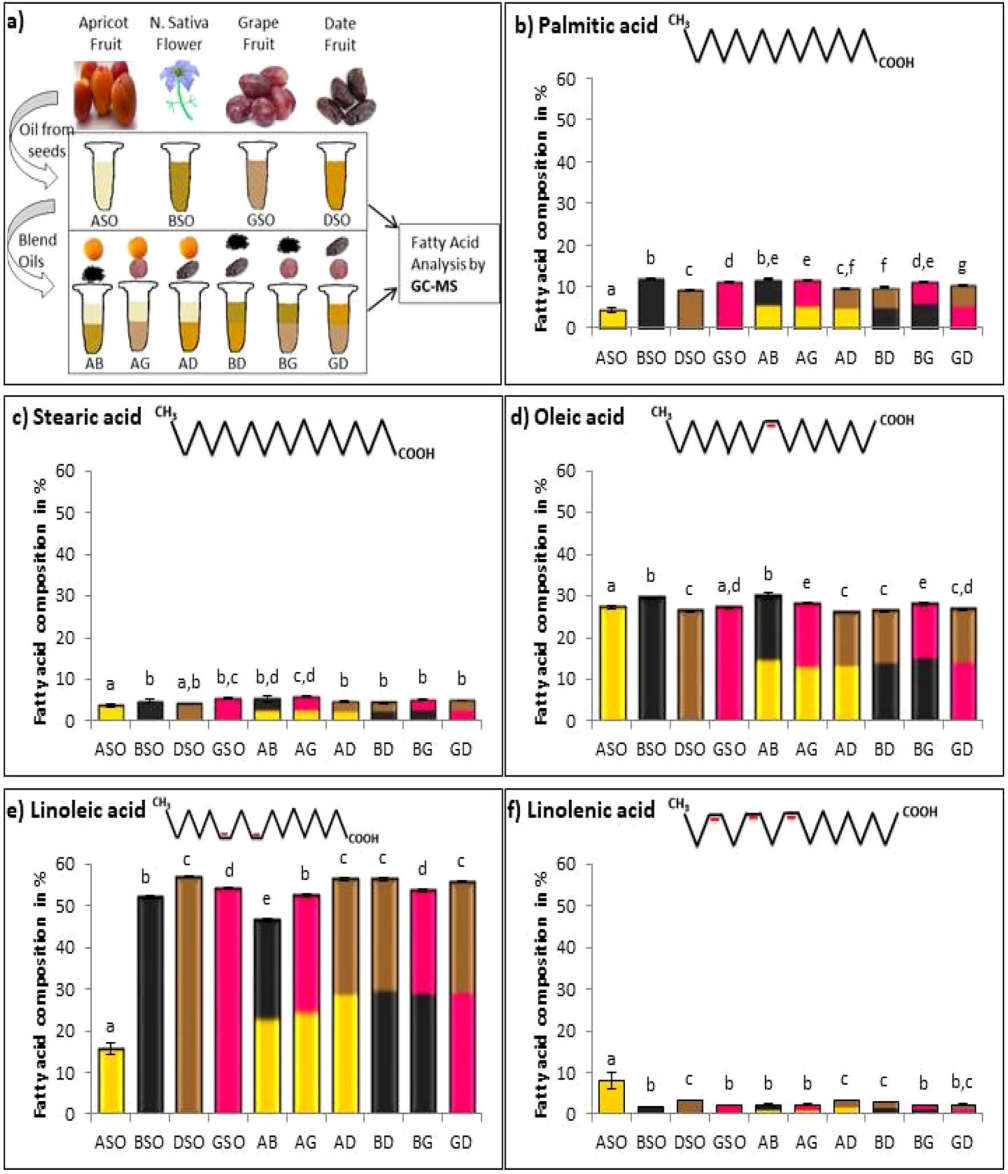
Fatty acid composition in oil samples in % reported as the mean ± standard deviation of three trials. Different letters indicate statistical significance (*p* < 0.05). ASO-Apricot seed oil, BSO-Black seed oil, DSO-Date seed oil, GSO-Grape seed oil, AB-ASO + BSO, AG-ASO + GSO, AD-ASO + DSO, BD-BSO + DSO, BG-BSO + GSO, GD-GSO + DSO.

Seed oils are rich in several important bioactive compounds including polyphenols, flavonoids, carotenoids, and fatty acids. Oils and oil mixtures were analyzed for their fatty acids (FA) composition (Fig. 1a). Unsaturated FA (oleic and linoleic acid) were dominating (up to 57%) compared to saturated FA (palmitic and stearic acid) (up to 12%). Fatty acid profiles of BSO, DSO, and GSO were similar (Fig. 1b–f). In these three oils, major FA found was linoleic acid (52%–57%, Fig. 1e) followed by oleic acid (26%–30%, Fig. 1d), palmitic acid (9%–12%, Fig. 1b), stearic acid (4%–5%, Fig. 1c), and finally linolenic acid (2%–3%, Fig. 1f).

Similar order of FA was reported for BSO and GSO^48,49^. Previous studies on DSO showed that oleic acid is the dominating FA^41,50^, while our results showed that linoleic acid is the dominating FA in this oil. This difference could be due to variations in cultivation practices and processing techniques of DSO^51^. ASO showed different percentages, where oleic acid was the major FA (27%), followed by linoleic acid (16%), linolenic acid (8%), palmitic acid (4%), and stearic acid (4%). ASO showed similar results in a previous study^12^. FA profile of oil mixtures was also rich in unsaturated FA with linoleic acid dominating in all samples. Blending oils showed significant changes in saturated and unsaturated FA in certain mixtures. Palmitic acid and oleic acid were significantly increased in AG compared to ASO and GSO alone. Similar findings were reported by Ramadan and coworkers, where they noticed an increase in palmitic acid and stearic acid when blending corn oil with black cumin seed oil^52^. In addition, Kaseke and coworkers reported similar results for oil blends^53^. On the other hand, some mixtures showed non-significant change compared to each oil alone. This was found for stearic acid in BD, BG, and GD, oleic acid in GD, and linolenic acid in BG and GD. Resulting amounts of fatty acids upon blending oils could be influenced by alterations in fatty acids, efficiency of oil blend, and matrix of oil mixtures^53^.

### Total phenolic content (TPC)

Oils are considered an important source of phenolic compounds^8^. However, their phenolic content is less than seed extracts, and this could be explained by the hydrophilic nature of phenolic compounds making them less soluble in the lipid fraction of the seed^16^.

**Figure 2:**
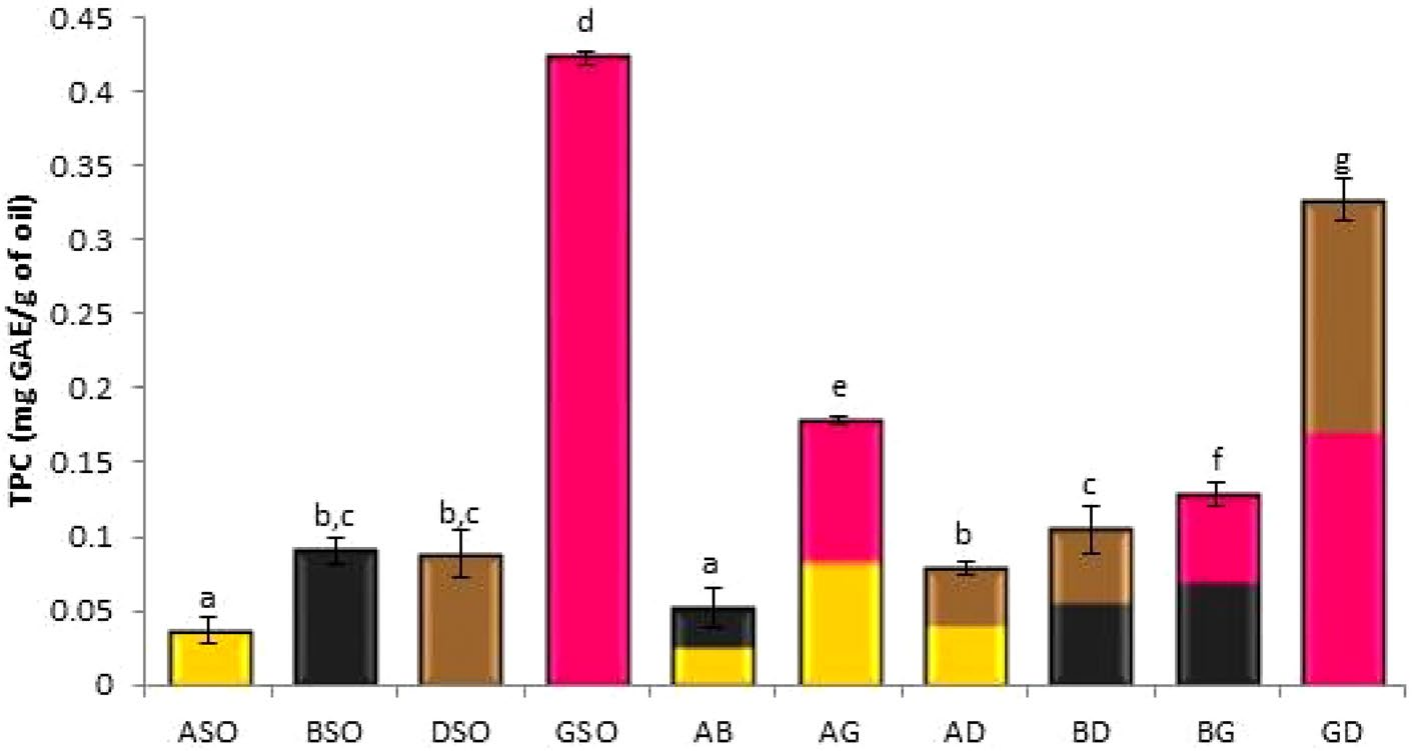
Total phenolic content (mg GAE/g of oil) reported as the mean ± standard deviation of three trials. Different letters indicate statistical significance (*p* < 0.05). ASO-Apricot seed oil, BSO-Black seed oil, DSO-Date seed oil, GSO-Grape seed oil, AB-ASO + BSO, AG-ASO + GSO, AD-ASO + DSO, BD-BSO + DSO, BG-BSO + GSO, GD-GSO + DSO.

Figure 2 shows total phenolic content (TPC) in oils and oil mixtures. GSO significantly showed the highest TPC (0.42 mg GAE/g of oil) while ASO significantly showed the lowest amount (0.03 mg GAE/g of oil). Similar polyphenol content were reported by^49,54^. BSO and DSO showed 0.09 and 0.08 mg GAE/g of oil, respectively with no significant difference between them. These results were slightly higher than those reported previously^41,48^. This could be due to differences in cultural conditions, extraction methods, and geographical locations^41^.

For mixtures, GD (0.32 mg GAE/g of oil) showed the highest phenolic content while AB (0.05 mg GAE/g of oil) showed the lowest. TPC of GSO was significantly higher than ASO, BSO, and DSO. Blending the oils increased TPC significantly up to four times in BG, AD, AG, and GD. Comparable results were reported by Kaseke and coworkers; where an increase in TPC content was found after blending sunflower oil with pomegranate seed oil^53^. BD showed non-significant difference in TPC compared to BSO and DSO.

### Antibacterial activity of single oils

**Figure 3:**
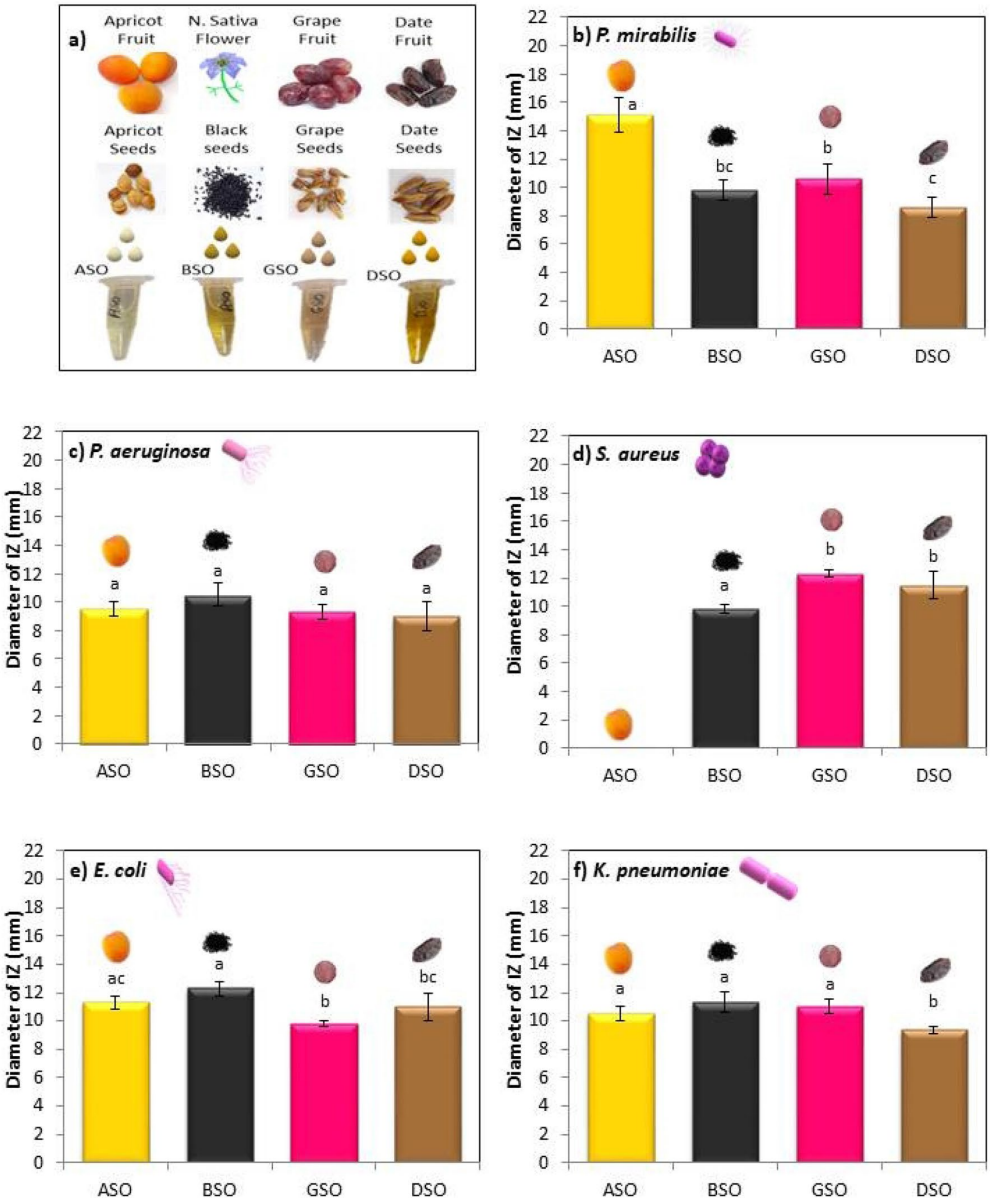
Antibacterial activity of (**a**) ASO, BSO, GSO and DSO against (**b**) *P. mirabilis*, (**c**) *P. aeruginosa*, (**d**) *S. aureus*, (**e**) *E. coli*, (**f**) *K. pneumoniae*, obtained by disk diffusion method. Results of inhibition zones (mm) are given as the mean ± standard deviation of three trials. Different letters indicate statistical significance (*p* < 0.05). ASO-Apricot seed oil, BSO-Black seed oil, DSO-Date seed oil, GSO-Grape seed oil.

Antibacterial activity of oils obtained from apricot seeds, black seeds, grape seeds, and date seeds (Fig. 3a) was evaluated against five bacterial strains using disk diffusion method (Fig. 3b–f). Against *P. mirabilis* (Fig. 3b), ASO showed significantly highest IZ (15.1 mm). Smaller IZs were observed for GSO (10.6 mm) followed by BSO (9.8 mm) with no significant difference between them (*p* > 0.05), and the lowest IZ was recorded for DSO (8.6 mm). On the other hand, no significant difference was found between tested oils against *P. aeruginosa* (Fig. 3c) (*p* > 0.05). IZs recorded against this strain were 10.5 mm for BSO, 9.5 mm for ASO, 9.3 mm for GSO, and 9 mm for DSO. Absence of inhibition was found only in ASO against *S. aureus* (Fig. 3d). GSO and DSO showed significantly the highest IZs of 12.3 mm and 11.5 mm respectively, with no significant difference between them, whereas DSO (9.83 mm) significantly showed the lowest IZ against *S. aureus*. While assessing the activity against *E. coli* (Fig. 3e), BSO significantly showed the highest IZ (12.3 mm) followed by ASO (11.3 mm) with no significant difference between them. Lower IZs were recorded for DSO (11.5 mm) and GSO (9.8 mm). While assessing the activity against *K. pneumoniae* (Fig. 3f), DSO significantly showed the lowest IZ of 9.33 mm. Higher IZs were obtained from BSO (11.33 mm), GSO (11 mm), and ASO (10.5 mm) with no observed significant difference between their zones.

The hydrophobic nature of oils facilitates their penetration through the lipid layers of bacterial cell membrane disturbing their structure, and thus increasing their permeability where ions and cell content can leak out^55^. Furthermore, antibacterial effect of the oil depends on the bacterial strain used as well as the oil tested where growth inhibition could be a result of synergistic interactions between certain components^56^.

ASO showed antibacterial effect against all strains, except for *S. aureus*. Limited studies were done on ASO. Previous studies focused on apricot’s seed extracts^13^ and essential oil^15^. Those studies showed antibacterial effect against similar strains but with higher activity, which agrees with those in earlier studies^57,58^, showing that essential oils and seed extracts could have higher antibacterial activity than the cold-pressed fixed oil extracted from the same seed. On the other hand, total absence of inhibition was found against *S. aureus* which could be because of the significant low amount of linoleic acid in ASO compared to the other oils. Important antibacterial activity was reported for linoleic acid against *S. aureus* by inhibiting its FabI, a protein responsible for fatty acid biosynthesis in bacteria^59^. It was also reported that when *S. aureus* is exposed to linoleic acid, fermentative and glycolic metabolic pathways are affected by altering their gene expression, leading to loss of its energy production^60^.

BSO showed an effect against all strains where *E. coli* had significantly the highest IZ. This is in agreement with previous studies where^61^ reported highest zone for *E. coli* compared to *S. aureus*. In addition, Arici and coworkers found also an effect for this oil against *K. pneumoniae* and *P. aeruginosa* along with the other strains; however, with higher IZs^62^. This could be explained by the fact that BSO bought from distinct commercial sources or extracted using different extraction methods from the same type of seeds have shown significant content variation in their major antibacterial chemical, thymoquinone^63^. Moreover, different storage conditions such as temperature and light could affect quinone content of the oil that is directly related to the antibacterial activity^63^.

GSO showed an effect against all the tested strains where *S. aureus* is significantly the most susceptible strain. This was in accordance with^64^, who reported the highest antibacterial effect of grape seed extract against *S. aureus*. More studies on grape seed extracts showed an effect against similar strains but with zones of inhibition higher than those produced in our study^20^. This is in accordance with the results of^58^ who found that GSO had the least antibacterial effect when they compared it with other different grape seed extracts. Studies conducted on GSO are limited. Among these studies,^65^ showed an antibacterial effect of GSO against *S. aureus* and *E. coli*.

DSO had shown inhibition against all the strains with zones ranging between 8.6 and 11.5 mm, where *S. aureus* and *E. coli* were significantly the most susceptible strains. This is in accordance with a study done by^25^ on the antibacterial effect of date seed extracts and found that *S. aureus* and *E. coli* were highly sensitive to extracts. Also, Aljazy *et al.* studied date seed extracts and found IZs^29^ against almost all the strains analyzed in our study, but with higher IZs that could be explained by the low solubility of polyphenols in oil^16^. However, only one study was found on the assessment of antibacterial effect of DSO, similarly showing an effect against *S. aureus* and *E. coli*^66^.

### Antibacterial activity of oils mixtures

**Figure 4:**
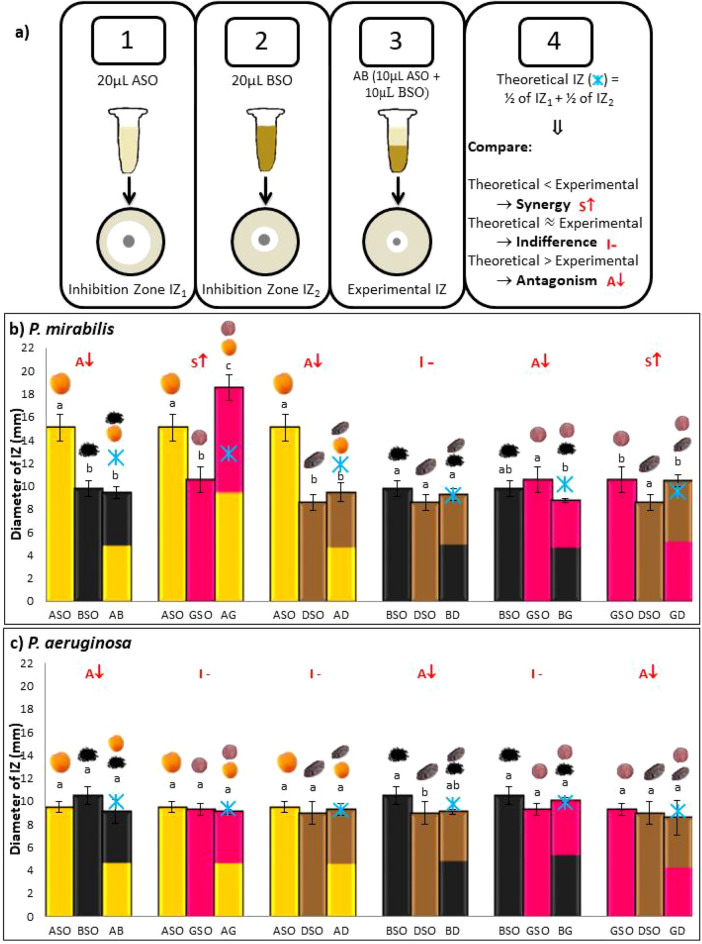
(**a**) Method illustration. (**b**, **c**) Theoretical IZ, experimental IZ, and interpreted effect of the antibacterial activity of AB, AG, AD, BD, BG, and GD mixtures obtained by disk diffusion method. Results of inhibition zones (mm) are given as the mean ± standard deviation of three trials. Different letters indicate statistical significance (*p* < 0.05). ASO-Apricot seed oil, BSO-Black seed oil, DSO-Date seed oil, GSO-Grape seed oil, AB-ASO + BSO, AG-ASO + GSO, AD-ASO + DSO, BD-BSO + DSO, BG-BSO + GSO, GD-GSO + DSO.

**Figure 5:**
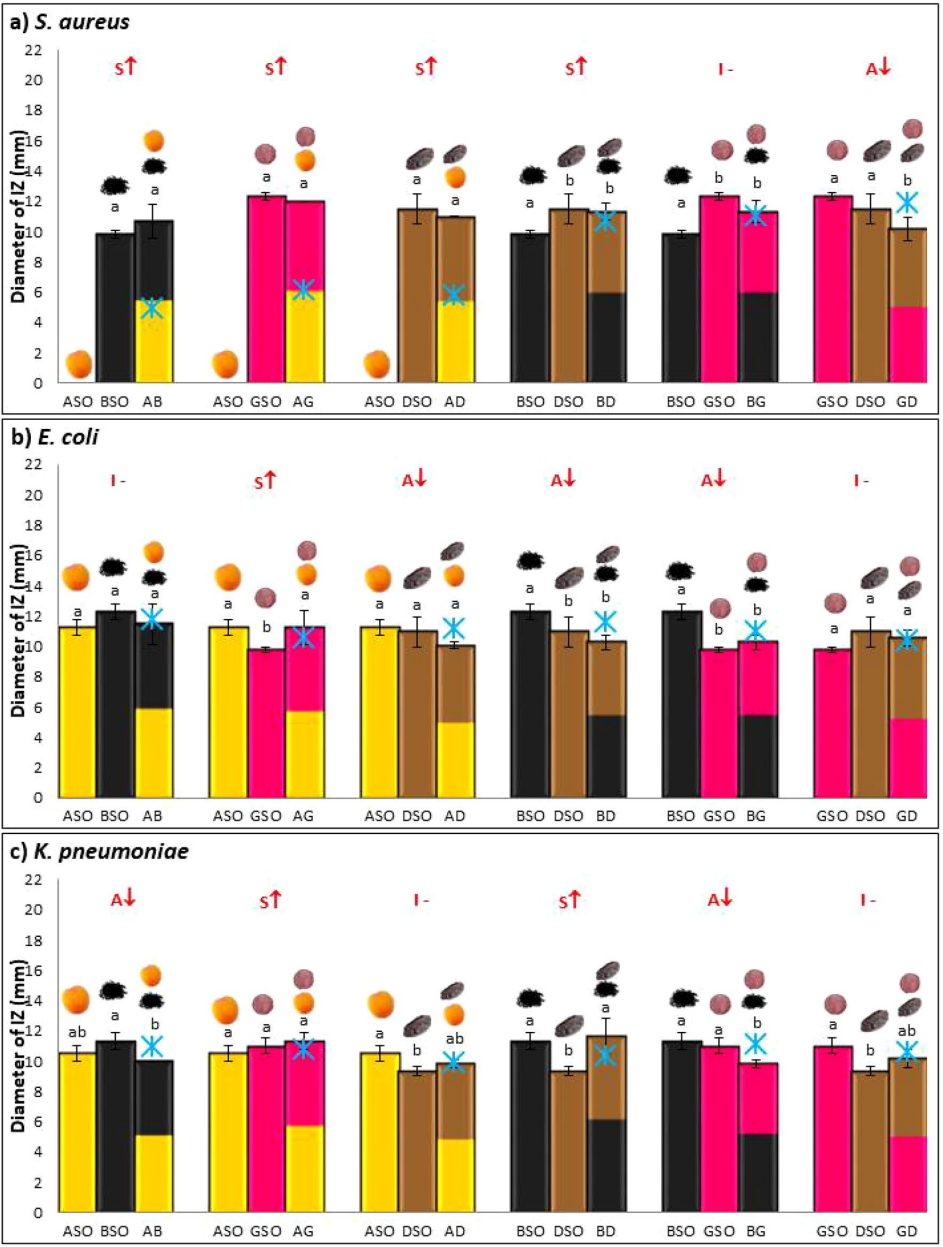
(**a**–**c**) Theoretical IZ, experimental IZ, and interpreted effect of the antibacterial activity of AB, AG, AD, BD, BG, and GD mixtures obtained by disk diffusion method. Results of inhibition zones (mm) are given as the mean ± standard deviation of three trials. Different letters indicate statistical significance (*p* < 0.05). ASO-Apricot seed oil, BSO-Black seed oil, DSO-Date seed oil, GSO-Grape seed oil, AB-ASO + BSO, AG-ASO + GSO, AD-ASO + DSO, BD-BSO + DSO, BG-BSO + GSO, GD-GSO + DSO.

Oils were mixed in binary mixtures and tested for possible synergistic antibacterial activity as mentioned in Fig. 4a. Figures 4b,c, 5a–c showed antibacterial activity of oil mixtures against each tested bacterial strain.

Experimental IZs of oil mixtures, theoretical IZs, as well as interpreted effects were reported. Type of interaction obtained when combining oils is a result of the interactions between their components^47^. Synergy occurs when a combination of two oils produces an antimicrobial effect greater than the sum of the individual oils. Antagonism occurs when the combination produces an antimicrobial effect lower than the sum of the individual oils. Indifference occurs when the combination produce an effect equal to the sum of the individual oils^67^.

Against *P. mirabilis*, synergistic interactions were found in AG and GD mixtures (Fig. 4b). The best effect was observed in AG mixture where its diameter of IZ measured 18.6 mm, while theoretically it should be 12.85 mm. Three antagonistic interactions were found in AB, AF, and BG mixture. One indifferent interaction was shown in BD mixture. A study was conducted on the antibacterial interaction of ferulic acid with other phenolic acids against *P. mirabilis*^68^. They found that combining caffeic acid and ferulic acid induce synergy against *P. mirabilis*; ferulic acid found in ASO, DSO, and GSO^31,69,70^ along with caffeic acid found in GSO and DSO^31,69^ could explain our results.

*P. aeruginosa* was the only strain that showed no synergism at all against all the mixtures tested (Fig. 4c). AB, BD, and GD showed antagonistic interaction while AG, AD, and BG showed indifferent interaction. Similar results were reported in previous studies when testing other oil mixtures against this strain^71,72^. This could be explained by the low permeability of its outer membrane. A study conducted by Mann and coworkers found that permeabilizing *P. aeruginosa*’s membrane increases the antibacterial activity of tea tree oil against it^73^.

ASO showed no antibacterial activity against *S. aureus*. However, when combined with BSO, GSO, and DSO an antibacterial activity was recorded (Fig. 5a). This could be explained by the increase in linoleic acid content of oil mixtures compared to ASO alone inducing an antibacterial activity against this strain. BD also showed synergy against *S. aureus* which could be due to gallic acid found also in DSO^26^ and thymol in BSO^36,74^. GD showed antagonistic effect while BG showed indifferent effect. Highest number of synergistic interactions was recorded against *S. aureus*, the only gram-positive strain investigated in this study. This could be due to the hydrophobic nature of oils that are not able to penetrate well through the outer hydrophilic membrane present in gram negative bacteria making them less susceptible to oils^75^.

One synergistic interaction was recorded for AG mixture against *E. coli* (Fig. 5b). This could be due to the interaction of gallic acid and ferulic acid found in both ASO and GSO^17,69,70^ that induces synergy once they are combined together^76^. Antagonism was found for AD, BD, and BG mixtures whereas indifference was found in GD and AB.

Against *K. pneumoniae*, synergy was found in AG and BD (Fig. 5c). Two antagonistic interactions in AB and BG, as well as two indifferent interactions in AD and GD were also recorded. Synergy in AG and BD could be due to poorly understood interactions between their components against *K. pneumoniae*, or due to synergistic interactions obtained in another gram negative bacteria belonging to the same family *Enterobacteriaceae*, *Salmonella *Typhi, after combining ferulic acid that is present in DSO and ASO^31,70^ with hydroxybenzoic acid found in BSO^77^ as well as caffeic acid found in GSO^68,69^.

AG showed synergistic interactions against all strains tested except *P. aeruginosa*. This is comparable to the results of FA and TPC where it shows a significant increase in two FA as well as TPC content. On the contrary, BG mixture was the only one with no synergistic interaction recorded. Overall, it is a complex mechanism when it comes to the antibacterial activity of single oils and oils mixtures against distinct bacterial strains. Different interactions between major and minor compounds, ability of compounds present in trace amount to alter activity of major ones, specificity of some compounds to certain bacteria, and susceptibility of tested bacterial strains can all influence the final effect produced^78^. More importantly, the cell penetration of different components, whether it is hydrophilic or lipophilic attraction, oils fixation on cell membranes and cell walls, and resistance mechanisms exerted by bacterial cells could strongly influence the type of interaction produced^79^. Hence, it is hard to predict what exact compounds are interacting together to produce synergistic or antagonistic antibacterial activity^78^.

## Conclusions

Using seed oils as antimicrobial agents is an important natural approach. This is also a remarkable way to utilize the oils extracted from fruit seeds instead of discarding them as industrial wastes. ASO and BSO were more effective against gram-negative bacteria while GSO and DSO were more effective against gram-positive bacterium. Blending oils improved FA profile of oils and their antibacterial activity. AG was the most efficient blend showing improved antibacterial activity. ASO showed the highest effect against *P. mirabilis*, and AG showed the highest number of synergistic interactions against all strains except *P. aeruginosa*. The results of this study showed that the oils studied have important antibacterial activity that was improved and strengthened when applied together in different mixtures. This was the first study revealing the nature of interactions of the antibacterial effect obtained from binary mixtures of ASO, DSO, GSO, and BSO. Further studies could be done to test their possible application as antibacterial compounds when applied using different methods in both in vivo and in vitro models.

## Data Availability

The data presented in this study are available on request from the corresponding author.

## References

[1] Kaseke T, Opara UL, Fawole OA (2020). Fatty acid composition, bioactive phytochemicals, antioxidant properties and oxidative stability of edible fruit seed oil: effect of preharvest and processing factors. Heliyon.

[2] Hassanien MMM (2014). Phytochemical contents and oxidative stability of oils from non-traditional sources. Eur. J. Lipid Sci. Technol..

[3] Fratianni F (2021). Fatty acid composition, antioxidant, and in vitro anti-inflammatory activity of five cold-pressed prunus seed oils, and their anti-biofilm effect against pathogenic bacteria. Front. Nutr..

[4] Thompson T (2022). The staggering death toll of drug-resistant bacteria. Nature.

[5] Rahman M, Sarker SD (2020). Antimicrobial natural products. Annu. Rep. Med. Chem..

[6] Zouhairi O, Saleh I, Alwan N, Toufeili I, Barbour E, Harakeh S (2012). Antimicrobial resistance of *Staphylococcus* species isolated from lebanese dairy-based products. East Mediterr Health J..

[7] Vaou N, Stavropoulou E, Voidarou C, Tsigalou C, Bezirtzoglou E (2021). Towards advances in medicinal plant antimicrobial activity: A review study on challenges and future perspectives. Microorganisms.

[8] Petropoulos SA (2021). Antimicrobial properties, cytotoxic effects, and fatty acids composition of vegetable oils from purslane, linseed, luffa, and pumpkin seeds. Appl. Sci..

[9] Baykara D, Pilavcı E, Meran M, Çalışkaner ZO (2021). Antimicrobial properties and application of fig seed oil as an additive for chitosan-based films. Ukr. Food J..

[10] Siddiqui SA, Anwar S, Yunusa BM, Nayik GA, Mousavi Khaneghah A (2023). The potential of apricot seed and oil as functional food: Composition, biological properties, health benefits & safety. Food Biosci..

[11] Saadi S (2022). Novel emulsifiers and stabilizers from apricot (*Prunus armeniaca* L.): Their potential therapeutic targets and functional properties. Appl. Food Res..

[12] Makrygiannis I (2023). Exploring the chemical composition and antioxidant properties of apricot kernel oil. Separations.

[13] Yiğit D, Yiğit N, Mavi A (2009). Antioxidant and antimicrobial activities of bitter and sweet apricot (*Prunus armeniaca* L.) kernels. Braz. J. Med. Biol. Res..

[14] Rai I, Bachheti RK, Saini CK, Joshi A, Satyan RS (2016). A review on phytochemical, biological screening and importance of Wild Apricot (*Prunus armeniaca* L.). Orient. Pharm. Exp. Med..

[15] Lee HH (2014). Chemical composition and antimicrobial activity of the essential oil of apricot seed. Phytother. Res..

[16] Garavaglia J, Markoski MM, Oliveira A, Marcadenti A (2016). Grape seed oil compounds: biological and chemical actions for health. Nutr. Metab. Insights.

[17] Yang C (2021). Processing technologies, phytochemical constituents, and biological activities of grape seed oil (GSO): A review. Trends Food Sci. Technol..

[18] Rombaut N (2014). Grape seed oil extraction: Interest of supercritical fluid extraction and gas-assisted mechanical extraction for enhancing polyphenol co-extraction in oil. Comptes. Rendus. Chim..

[19] Martin ME, Grao-Cruces E, Millan-Linares MC, Montserrat-De la Paz S (2020). Grape (*Vitis vinifera* L.) seed oil: A functional food from the winemaking industry. Foods.

[20] Kandasamy M (2016). A study on antibacterial effect of grape seed extracts in common clinical and drug resistant isolates. Int. J. Clin. Trials.

[21] Eslami H (2017). Evaluation of antifungal effect of grape seed extract (Gse) on candida glabrata and candida krusei: In vitro study. Biomed. Res..

[22] Ismail AFM, Salem AAM, Eassawy MMT (2016). Hepatoprotective effect of grape seed oil against carbon tetrachloride induced oxidative stress in liver of γ-irradiated rat. J. Photochem. Photobiol. B Biol..

[23] Ismail AFM, Moawed FSM, Mohamed MA (2015). Protective mechanism of grape seed oil on carbon tetrachloride-induced brain damage in γ-irradiated rats. J. Photochem. Photobiol. B: Biol..

[24] Zhao L (2015). Muscadine grape seed oil as a novel source of tocotrienols to reduce adipogenesis and adipocyte inflammation. Food Funct..

[25] Perveen K, Bokahri NA (2020). Comparative analysis of chemical, mineral and in-vitro antibacterial activity of different varieties of date fruits from Saudi Arabia. Saudi J. Biol. Sci..

[26] Mrabet A, Jiménez-Araujo A, Guillén-Bejarano R, Rodríguez-Arcos R, Sindic M (2020). Date seeds: A promising source of oil with functional properties. Foods.

[27] Bouhlali E (2017). Phytochemical compositions and antioxidant capacity of three date (*Phoenix dactylifera* L.) seeds varieties grown in the South East Morocco. J. Saudi Soc. Agric. Sci..

[28] Nehdi I, Omri S, Khalil MI, Al-Resayes SI (2010). Characteristics and chemical composition of date palm (*Phoenix canariensis*) seeds and seed oil. Ind. Crops Prod..

[29] Aljazy NA, Al-Mossawi AEBH, Al-Rikabi AK (2019). Study of antibacterial activity of some date seed extracts. Basrah J. Agric. Sci..

[30] Metoui M, Essid A, Bouzoumita A, Ferchichi A (2019). Chemical composition, antioxidant and antibacterial activity of Tunisian date palm seed. Polish J. Environ. Stud..

[31] Harkat H (2022). Assessment of biochemical composition and antioxidant properties of Algerian date palm (*Phoenix dactylifera* L.) seed oil. Plants.

[32] Ines D (2010). Date seed oil inhibits Hydrogen peroxide-induced oxidative stress in human epidermal keratinocytes. Int. J. Dermatol..

[33] Dammak I, Boudaya S, Ben Abdallah F, Turki H, Attia H (2010). Effect of date seed oil on p53 expression in normal human skin. Connect. Tissue Res..

[34] Forouzanfar F, Fazly Bazzaz BS, Hosseinzadeh H (2014). Black cumin (*Nigella sativa*) and its constituent (thymoquinone): A review on antimicrobial effects. Iran. J. Basic Med. Sci..

[35] Mahboubi M (2018). Natural therapeutic approach of *Nigella sativa* (Black seed) fixed oil in management of Sinusitis. Integr. Med. Res..

[36] Mohammed, S. J. *et al.* Structural Characterization, Antimicrobial Activity, and in Vitro Cytotoxicity Effect of Black Seed Oil. *Evidence-based Complement. Altern. Med.* **2019**, (2019). 10.1155/2019/6515671 PMC6721493 31531117

[37] Warinhomhoun S (2023). Effects of black seed oil combined with olive oil or honey on antioxidant activities, phenolic content, and identification and quantification of thymoquinone, a key bioactive compound. J. Agric. Food Res..

[38] Barakat EMF, El Wakeel LM, Hagag RS (2013). Effects of *Nigella sativa* on outcome of hepatitis C in Egypt. World J. Gastroenterol..

[39] Suri K, Singh B, Kaur A, Yadav MP (2023). Physicochemical characteristics, oxidative stability, pigments, fatty acid profile and antioxidant properties of co-pressed oil from blends of peanuts, flaxseed and black cumin seeds. Food Chem. Adv..

[40] Basavegowda N, Baek KH (2021). Synergistic antioxidant and antibacterial advantages of essential oils for food packaging applications. Biomolecules.

[41] Ourradi H (2021). Proximate composition of polyphenolic, phytochemical, antioxidant activity content and lipid profiles of date palm seeds oils (*Phoenix dactylifera* L.). J. Agric. Food Res..

[42] Abdullah F, Ismail R, Ghazali R, Idris Z (2018). Total phenolic contents and antioxidant activity of palm oils and palm kernel oils at various refining processes. J. Oil Palm Res..

[43] Graczyk F, Strzemski M, Balcerek M, Kozłowska W, Mazurek B, Karakuła M, Załuski D (2021). Pharmacognostic evaluation and HPLC–PDA and HS–SPME/GC–MS metabolomic profiling of eleutherococcus senticosus fruits. Molecules.

[44] He M, Li Y, Yan J, Cao D, Liang Y (2013). Analysis of essential oils and fatty acids from platycodi radix using chemometric methods and retention indices. J. Chromatogr. Sci..

[45] Poonkothai M, Saravanan M (2008). Antibacterial activity of *Aegle marmelos* against leaf, bark and fruit extracts. Anc. Sci. Life.

[46] Bekka-Hadji F, Bombarda I, Djoudi F, Bakour S, Touati A (2022). Chemical composition and synergistic potential of *Mentha pulegium* L. and Artemisia herba alba Asso. Essential oils and antibiotic against multi-drug resistant bacteria. Molecules.

[47] Kon K, Rai M (2012). Antibacterial activity of Thymus vulgaris essential oil alone and in combination with other essential oils..

[48] Kiralan M, Özkan G, Bayrak A, Ramadan MF (2014). Physicochemical properties and stability of black cumin (*Nigella sativa*) seed oil as affected by different extraction methods. Ind. Crops Prod..

[49] Konuskan DB, Kamiloglu O, Demirkeser O (2019). Fatty acid composition, total phenolic content and antioxidant activity of grape seed oils obtained by cold- pressed and solvent extraction. Indian J. Pharm. Educ. Res..

[50] Tafti G, Dahdivan S, Ardakani Y (2017). Physicochemical properties and applications of date seed and its oil. Int. Food Res. J..

[51] Coughlan, R., Moane, S. & Larkin, T. Variability of essential and nonessential fatty acids of irish rapeseed oils as an indicator of nutritional quality. *Int. J. Food Sci.* **2022**, (2022). 10.1155/2022/7934565 PMC8769870 35071588

[52] Ramadan MF, Wahdan KMM (2012). Blending of corn oil with black cumin (*Nigella sativa*) and coriander (*Coriandrum sativum*) seed oils: Impact on functionality, stability and radical scavenging activity. Food Chem..

[53] Kaseke T, Opara UL, Fawole OA (2021). Blending of sunflower oil with pomegranate seed oil from blanched seeds: Impact on functionality, oxidative stability, and antioxidant properties. Processes.

[54] Uluata S (2016). Effect of extraction method on biochemical properties and oxidative stability of apricot seed oil. Akad. Gıda.

[55] Burt S (2004). Essential oils: Their antibacterial properties and potential applications in foods—a review. Int. J. Food Microbiol..

[56] Larif M, Ouhssine M, Soulaymani A, Elmidaoui A (2013). Potential effluent oil mills and antibacterial activity polyphenols against some pathogenic strains. Res. Chem. Intermed..

[57] Aydeniz Güneşer B, Demirel Zorba NN, Yılmaz E (2018). Antimicrobial activity of cold pressed citrus seeds oils, some citrus flavonoids and phenolic acids. Riv. Ital. delle Sostanze Grasse.

[58] Yong-heng L (2020). Antibacterial activity of grape seed extracts and chemical components analysis of grape seed oil. Sci. Technol. Food Ind..

[59] Zheng CJ (2005). Fatty acid synthesis is a target for antibacterial activity of unsaturated fatty acids. FEBS Lett..

[60] Kenny JG (2009). The staphylococcus aureus response to unsaturated long chain free fatty acids: Survival mechanisms and virulence implications. PLoS One.

[61] Sivagurunathan Moni S, Sivakumar S (2014). Antibacterial spectrum of black seed oil against selected human pathogenic bacteria. J. Pharm. Res..

[62] Arici M, Sagdic O, Gecgel U (2005). Antibacterial effect of Turkish black cumin (*Nigella sativa* L.) oils. Grasas y Aceites.

[63] Khan AR, Kour K (2016). Wide spectrum antibacterial activity of *Nigella sativa* L. seeds. IOSR J. Pharm..

[64] Kao TT (2010). Grape seed extract inhibits the growth and pathogenicity of Staphylococcus aureus by interfering with dihydrofolate reductase activity and folate-mediated one-carbon metabolism. Int. J. Food Microbiol..

[65] Khah MD, Ghanbarzadeh B, Roufegarinejad Nezhad L, Ostadrahimi A (2021). Effects of virgin olive oil and grape seed oil on physicochemical and antimicrobial properties of pectin-gelatin blend emulsified films. Int. J. Biol. Macromol..

[66] Ekpa OD, Ebana RUB (1996). Comparative studies of mmanyanga, palm and coconut oils: antimicrobial effects of the oils and their metallic soaps on some bacteria and fungi. Global J. Pure Appl. Sci..

[67] Hyldgaard M, Mygind T, Meyer RL (2012). Essential oils in food preservation: Mode of action, synergies, and interactions with food matrix components. Front. Microbiol..

[68] Ogunsina O, Olabode Isaiah O (2020). The synergistic interaction of phenolic compounds in pearl millets with respect to antioxidant and antimicrobial properties antioxidant and nutritive value of fermented melon (*Ogiri Egusi*). View project evaluation of heavy metals in selected smoked meat in Uso and Owena markets, Nigeria view project. Am. J. Food Sci. Heal..

[69] Pérez C, Ruiz Del Castillo ML, Gil C, Blanch GP, Flores G (2015). Supercritical fluid extraction of grape seeds: extract chemical composition, antioxidant activity and inhibition of nitrite production in LPS-stimulated Raw 264.7 cells. Food Funct..

[70] Hrichi S, Rigano F, Chaabane-Banaoues R, Oulad El Majdoub Y, Mangraviti D, Di Marco D, Cacciola F (2020). Identification of fatty acid, lipid and polyphenol compounds from *Prunus armeniaca* L. kernel extracts. Foods.

[71] Reza Heidari-Soureshjani, R. A. *et al.* Study of the bactericidal and bacteriostatic effects of olive oil, sesame oil and their synergism on Pseudomonas aeruginosa in vitro. http://futurenatprod.skums.ac.ir/article_35195.html (2016).

[72] Semeniuc CA, Pop CR, Rotar AM (2017). Antibacterial activity and interactions of plant essential oil combinations against Gram-positive and Gram-negative bacteria. J. Food Drug Anal..

[73] Mann CM, Cox SD, Markham JL (2000). The outer membrane of *Pseudomonas aeruginosa* NCTC 6749 contributes to its tolerance to the essential oil of Melaleuca alternifolia (tea tree oil). Lett. Appl. Microbiol..

[74] Zhang X (2022). Synergistic inactivation of *Escherichia coli* O157:H7 and Staphylococcus aureus by gallic acid and thymol and its potential application on fresh-cut tomatoes. Food Microbiol..

[75] Al-Bayati FA (2008). Synergistic antibacterial activity between *Thymus vulgaris* and *Pimpinella anisum* essential oils and methanol extracts. J. Ethnopharmacol..

[76] de Oliveira EF, Nguyen CH, Stepanian K, Cossu A, Nitin N (2019). Enhanced bacterial inactivation in apple juice by synergistic interactions between phenolic acids and mild food processing technologies. Innov. Food Sci. Emerg. Technol..

[77] Zarrouk A (2019). Profile of Fatty acids, tocopherols, phytosterols and polyphenols in mediterranean oils (Argan oils, olive oils, milk thistle seed oils and nigella seed oil) and evaluation of their antioxidant and cytoprotective activities. Curr. Pharm. Des..

[78] Ndayishimiye J, Lim DJ, Chun BS (2018). Antioxidant and antimicrobial activity of oils obtained from a mixture of citrus by-products using a modified supercritical carbon dioxide. J. Ind. Eng. Chem..

[79] Bakkali F, Averbeck S, Averbeck D, Idaomar M (2008). Biological effects of essential oils – A review. Food Chem. Toxicol..

